# A Molecular Integrative Study on the Inhibitory Effects of WRR and ERW on Amyloid β Peptide (1–42) Polymerization and Cell Toxicity

**DOI:** 10.3390/polym15224356

**Published:** 2023-11-08

**Authors:** Zhongyun Wu, Lianmeng Ye, Nan Yuan, Nuela Manka’a Che Ajuyo, Zhengpan Xiao, Liangwang Liu, Zuqian Chen, Yechun Pei, Yi Min, Dayong Wang

**Affiliations:** 1 Laboratory of Biopharmaceuticals and Molecular Pharmacology, School of Pharmaceutical Sciences, Hainan University, Haikou 570228, China; 2 Department of Biotechnology, School of Life Sciences, Hainan University, Haikou 570228, China; 3 One Health Cooperative Innovation Center, Hainan University, Haikou 570228, China; 4 Key Laboratory of Tropical Biological Resources of the Ministry of Education of China, Hainan University, Haikou 570228, China

**Keywords:** Alzheimer’s disease, Aβ42, polymerization, inhibitors, molecular docking, molecular dynamics, WRR, ERW

## Abstract

Alzheimer’s disease (AD) is a neurodegenerative disease and the main pathological characteristic of AD is the deposition of Aβ42 in the brain. Inhibition of Aβ42 polymerization is one of the important research directions. Due to the pathological complexity of Alzheimer’s disease, studies on Aβ42 polymerization inhibitors have not made significant progress worldwide. Using an independently constructed structure database of oligopeptides, in this study, molecular docking, umbrella sampling analysis of free energy, ThT fluorescence detection of Aβ42 polymerization, transmission electron microscopy, and flow cytometry detection of reactive oxygen species (ROS) and apoptosis were performed to screen tripeptides and pentapeptides that inhibit polymerization. It was found that two tripeptides, i.e., WRR and ERW, bind stably to the core of Aβ42 polymerization in the molecular dynamics analysis, and they significantly inhibited the aggregation of Aβ42 and reduced their cell toxicity in vitro.

## 1. Introduction

Alzheimer’s disease (AD) is one of the top ten causes of death in the United States. Its main pathological characteristic is extracellular plaques made of amyloid β peptide (Aβ) and neurofibrillary tangles [1,2]. The aggregation of Aβ is considered to play a key role in the pathogenesis of AD [3,4]. Amyloid precursor protein (APP) is hydrolyzed by β-secretion enzymes and γ-secretion enzymes to produce Aβ [5]. Aβ40 and Aβ42 are the two common species of Aβ. Among some patients with familial AD, an increase in the total level of Aβ or an increase in the ratio of Aβ42/Aβ40 has been observed [6,7,8]. In the human brain, due to continuous sedimentation of Aβ42, a certain magnitude of its accumulation can convert the solubilized Aβ42 monomers clusters into a gathering body of polymers of varying degrees, which cause various pathological changes [9,10,11]. At present, Aduhelm and Lecanemab, which are monoclonal antibodies approved by the FDA, are the only medicines that target amyloid β peptide (Aβ) for Alzheimer’s disease.

Through phage display, Kawasaki et al. found that short peptides could bind to soluble Aβ42 and inhibit the formation of oligomers, and they preliminarily verified that some tripeptides containing arginine could inhibit the formation of Aβ42 aggregates [12,13]. In recent years, computer-aided drug design has been successful in designing various medicines [14]. Additionally, based on the results of molecular docking and dynamics analyses, we have found that dipeptides (arginine-arginine, R-R) form hydrogen bonds with the backbone atoms of Aβ42 oligomers to inhibit their elongating into fibrils [15]. Thus, we suspected that arginine tripeptide had inhibitory activity on the formation of Aβ42 aggregates. Computer-aided drug design and in vitro pharmacological screening approaches were adopted in this study. First, an oligopeptide structure database was constructed and molecular docking was performed, then and a molecular dynamics (MD) analysis, such as the umbrella sampling of binding free energy, was performed to analyze the binding of oligopeptides to Aβ42. Based on the MD results, THT fluorescence detection of Aβ42 polymerization, electron microscopy, and the flow cytometry detection of reactive oxygen species (ROS) and apoptosis in the cells secreting Aβ42 were conducted. The purpose of this study was to screen oligopeptides that inhibit polymerization of Aβ42 and reduce the cytotoxicity induced by secreted Aβ42 from the library of peptides composed of the 20 common L-α-amino acids.

## 2. Materials and Methods

### 2.1. Materials

WRR and ERW were synthesized by Sangon Biotech (Shanghai, China), with a purity of more than 98%, and dissolved in 10 mM phosphate buffer (pH 7.4). Aβ42 was purchased from ChinaPeptides (QYAOBIO) (Shanghai, China), and Aβ42 powder was dissolved to 1 mM with DMSO. Aliquots of Aβ42 and oligopeptides solutions were stored at −80 °C until use. DMSO, ThT, and Lipofectamine2000 were purchased from Sigma-Aldrich (St. Louis, MI, USA). The ROS active oxygen testing kit and the apoptosis kit for the detection by flow cytometry were purchased from Bioscience (Shanghai, China). PEI 40 K transfection reagents were purchased from Servicebio (Wuhan, China).

### 2.2. Molecular Docking

The structure database of oligopeptides consisting of the 20 L-α amino acids was constructed using Python 3.2.2 by calling the ChemScript module of the ChemDraw software 2020 (Providence, RI, USA). The Aβ42 structural data file (PDB# 5OQV) was downloaded from the Protein Data Bank (PDB). The structural data were corrected, and missing hydrogen atoms were added. The open-source software AutoDock 4 was used to predict the binding of oligopeptides to Aβ42, using the Amber99 force field. The binding sites and conformations of oligopeptides were initially screened using the London algorithm for free energy, and then refined using the generalized Born volume integral/weighted surface area algorithm.

### 2.3. Umbrella Sampling of Binding Free Energy

Binding free energy is reflected in the change in Gibbs free energy (∆G) in an isothermal-isobaric ensemble during the process of pulling oligopeptides away from Aβ42. The Aβ42 protein complex attached to the dipeptide was centered at 3.0, 3.5, and 1.5 (x, y, and z) nm in a unit cell with periodic boundaries, which had dimensions of 6.0, 7.0, and 14.0 (x, y, and z) nm. Water molecules were introduced into the cell, and sodium chloride at a final concentration of 0.1 M was used to neutralize the system. The carbonyl carbon atom (Cα) of the 29th glycine and the Cα of an oligopeptides’ second amino acid were chosen as reference atoms. The pressure was equilibrated before the pulling and umbrella sampling procedures. The two proteins were pulled apart at a constant speed of 0.01 nm/ps to generate a series of configurations by applying a harmonic force, and 501 coordinate files were saved during the pulling process. Then, in overlapping 0.2-nm-spacing sampling windows along the reaction axis (ξ), from 23 to 25 umbrella samplings of 10 ns each were performed, generating roughly 450 Gb of data. The ∆G was estimated using GROMACS’s WHAM module (2020.3).

### 2.4. ThT Fluorescent Detection of Aggregation of Aβ42

ThT was dissolved in 10 mM of PBS to make a stocking solution and filtrated through the 0.22 μm filter. The final concentration of Aβ42 was 10 μM, the final concentration of the tripeptides, i.e., WRR and ERW, was 40 μM, and the final concentration of ThT was 50 μM. The samples and reagents were added to black 96-well plates, and the total volume of a sample was 120 μL per well, with each sample setup consisting of five replications. Then, the samples were incubated at 37 °C for 48 h while shaking. The fluorescence was detected at 485 nm at an interval of 5 min with an exciting wavelength of 450 nm.

### 2.5. Transmission Electron Microscopy Observations of Aggregated Aβ42

The aggregation morphology of Aβ42 was observed using a transmission electron microscope (TEM). The experimental group Aβ42 was incubated with either WRR or ERW. The final concentration of Aβ42 was 10 μM, and that of either WRR or ERW was 40 μM. After incubated at 37 °C for 48 h, 10 μL samples were dropped onto carbon film-supported copper grids. The samples were negatively stained with 10 μL of 2% phosphotungstic acid solution. After drying, the samples were observed using a transmission electron microscope with an accelerating voltage of 200 kV.

### 2.6. SH-SY5Y Cell Culture

SH-SY5Y cells removed from the liquid nitrogen were rapidly thawed at 37 °C. After re-suspension in DMEM containing 5% fetal bovine serum and antibiotics, the cells were transferred to cell culture dishes coated with the gelatin made from deep-sea fish skin, and then cultured in an CO_2_ incubator at 37 °C. When the cultured cells reached 80% of confluency, they were used for experiments.

### 2.7. Transfection and Expression of Secreted Aβ42

Before transfection, the medium was replaced with Opti-MEM and cultured for 2 h. Then, the cells were transfected with pcDNA3.1-Aβ42 plasmids for 4 h, in which the sequence encoding the signal peptide for secretion had been added to the 5′ end of Aβ42. Four hours after transfection, it was switched to the DMEM medium containing 5% FBS, and 24 h after transfection, the antibiotics were added to the culture.

### 2.8. Flow Cytometry Methods to Detect Reactive Oxygen Species and Apoptosis

Either WRR or ERW at final concentrations of 10 μM and 50 μM were added to the cultured cells expression secreted Aβ42 at 37 °C for 24 h. Then, reactive oxygen species (ROS) and cell apoptosis kits (UElandy Inc., Suzhou, China) were used for detection on a CytoFlex LX flow cytometer (Beckman, Brea, CA, USA).

## 3. Results

### 3.1. Molecular Docking of Tripeptides

**Figure 1:**
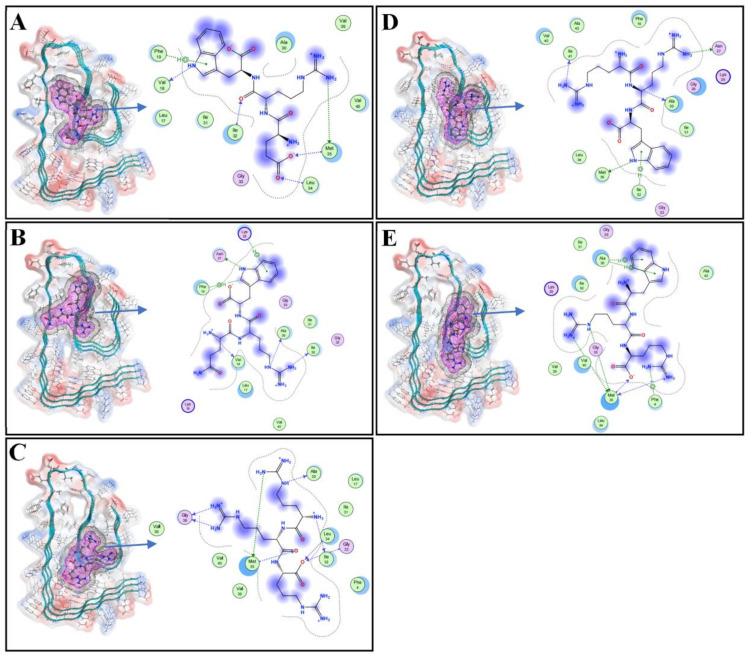
Three-dimensional docking diagram of tripeptides with Aβ42: (**A**) Binding of ERW with Aβ42; (**B**) binding of QRW with Aβ42; (**C**) binding of RRR with Aβ42; (**D**) binding of RRW with Aβ42; (**E**) binding of WRR with Aβ42. For clarity, only three strands taken from Aβ42 pentamer are shown in the figures. The red mesh represents the molecular surfaces of the tripeptides, and the gray mesh represents the boundaries of van der Waal’s force.

The conformational change of Aβ42 was mainly affected by hydrophobic interaction and electrostatic forces including hydrogen bonds [16]. The binding of Aβ42 with a ligand may effectively prevent the generation of hydrogen bonds between Aβ42 and Aβ42, and therefore inhibit polymerization of Aβ42 [17]. Our earlier article demonstrated that three hydrophobic clusters produced in Aβ42 polymers are beneficial to the binding energy between Aβ42 strands, with the first hydrophobic cluster being composed of the N-terminal 2nd alanine, 4th phenylalanine, 34th leucine, and 36th valine. The 17th leucine, 19th phenylalanine, 21st alanine, 24th valine, and 31st isoleucine form the second hydrophobic cluster. The third hydrophobic cluster is formed by the 30th alanine, 32nd isoleucine, 35th methionine, 40th valine, and 42nd alanine at the C-terminus [15]. Polymerized Aβ42 adopts a conformation in which the backbone atoms are distributed almost in a flat plane, while the Aβ42 monomer is a globular protein, in the MD analysis. Previous molecular docking studies have found that a few dipeptides bonded to the polymerization core of Aβ42, and thus inhibited polymerization [15]. In the present study, a molecular docking experiment between Aβ42 and oligopeptides was carried out. Five tripeptide compounds were screened in the Aβ42 hydrophobic cluster, as shown in Figure 1, namely glutamate-arginine-tryptophan (ERW), glutamine-arginine-tryptophan (QRW), arginine-arginine-arginine (RRR), arginine-arginine-tryptophan (RRW), and tryptophane-arginine-arginine (WRR). Figure 1 shows the binding of tripeptides to Aβ42, where the magenta grid is the molecular surface of the tripeptide, and the gray grid is the range of van der Waals forces. The Aβ42 residues involved in the ERW hydrogen bond interaction are Val18, Phe19, IIe32, Leu34, Met35; the Aβ42 residues involved in the interaction between QRW and Aβ42 are Val18, Phe19, Asn27, Lys28, Ala30, and IIe32; the Aβ42 residues involved in the interaction between RRR and Aβ42 are Ala30, IIe32, Gly33, Leu34, Met35, and Gly38; the Aβ42 residues involved in the interaction between RRW and Aβ42 are Asn27, Ala30, IIe32, Met35, IIe41; the Aβ42 residues involved in the hydrogen bond interaction between WRR and Aβ42 are 4Phe, 30Ala, and 35Met. The interactions between Aβ42 and the tripeptide ligands in Figure 1 show that ERW can bind to phenylalanine 19, isoleucine 32, and methionine 35 of the amino acid residues of Aβ42 hydrophobic clusters through hydrogen bonds including π-type bonding. WRR can interact with phenylalanine 4, alanine 30, and methionine 35 of Aβ42 hydrophobic cluster amino acid residues. These results suggest that the polymerization of Aβ42 may be effectively inhibited by ERW and WRR.

### 3.2. Evaluation of Conformational Rationality Using a Ramachandran Plot

**Figure 2:**
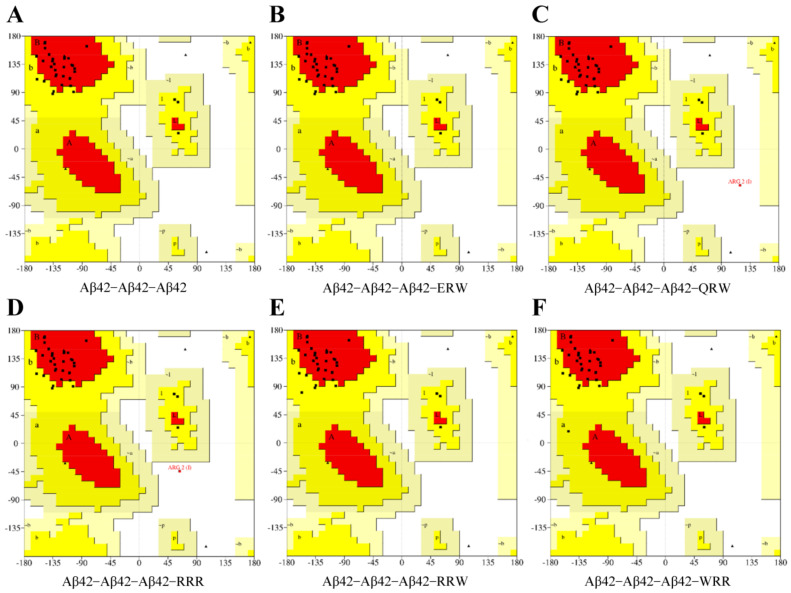
Ramachandran plot of Aβ42 and tripeptide complexes: (**A**) Aβ42−Aβ42 dimer; (**B**) Aβ42–ERW binding complex; (**C**) Aβ42–QRW binding complex; (**D**) Aβ42–RRR binding complex; (**E**) Aβ42–RRW binding complex; (**F**) Aβ42–WRR binding complex.

A Ramachandran plot describes the peptide main backbone dihedral angles φ and ψ in the context of their principal degrees of freedom. The method was used to assess conformational rationality of protein complexes, and minimal differences were observed between experimental and simulated results [18,19,20]. The Ramachandran plot shown in Figure 2 is color coded, with black squares representing highly preferred observations, red squares representing preferred observations, and black triangles representing suspicious observations. In general, if the amino acid residues in the allowable (yellow) and maximum allowable (red) regions account for more than 90% of the whole protein, and the number of suspicious observations is less than 5% of the total amino acids in the protein, we can consider that the conformation of the model accords with the rules of stereochemistry. The Aβ42–WRR–Aβ42 complex (Figure 2F) has the highest highly preferred value, that is, the number of highly preferred amino acids accounted for 83.951% of the total number of amino acids, and the suspicious impermissible region of Aβ42–WRR–Aβ42 accounted for only 3.704%; the region of the Aβ42–ERW–Aβ42 complex (Figure 2C) with the second highest optimal value accounted for 81.481% and 4.938%, respectively, and was located in the suspected disallowed region. The results indicated that the conformation of the complex formed by the interaction between the two tripeptide compounds and Aβ42 was reasonable. It is suggested that either WRR or ERW can be well combined into Aβ42 aggregates and may play a potential inhibitory effect on Aβ42 aggregation.

### 3.3. Umbrella Sampling Analysis of Ligand and Aβ42 Monomer

**Figure 3:**
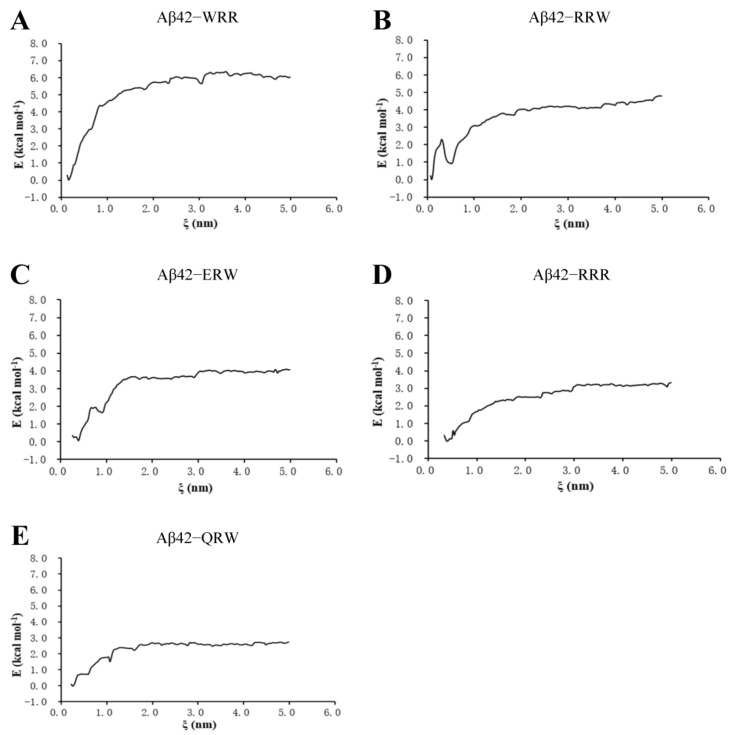
Umbrella sample of binding free energy of tripeptides with Aβ42: (**A**) WRR and Aβ42; (**B**) RRW and Aβ42; (**C**) ERW and Aβ42; (**D**) RRR and Aβ42; (**E**) QRW and Aβ42. ξ, the reaction axis.

In order to solve the weakness associated with molecular docking, which was the lack of time dimension and the indirect calculation of the solvent effects, the MD analysis was conducted. In the umbrella sampling of binding free energy, a harmonic force was applied to pull oligopeptides from the core of the polymerization of Aβ42, and the system configurations with a center distance less than 0.2 nm were manually selected as sampling windows for Gibbs free energy which reflects the change of protein–ligand binding free energy [21]. The ∆G along the reaction axis, ξ, during separation is shown in Figure 3. For the Aβ42–WRR complex, it induced the highest change of free energy to separate WRR from Aβ42 among the five tripeptides, i.e., about 6.0 kcal/mol (Figure 3A), indicating that WRR binds to Aβ42 the strongest and has a potential inhibition effect on Aβ42 polymerization. The binding free energy for the Aβ42–ERW complex was about 4.0 kcal/mol (Figure 3C).

### 3.4. Effects of Oligopeptides on Aggregation of Aβ42 Detected by ThT Fluorescence Assay

**Figure 4:**
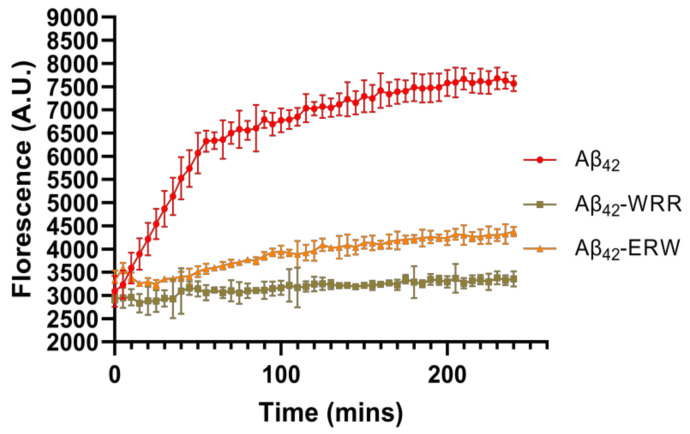
ThT fluorescence intensity of Aβ42 incubated with or without WRR and EWR. Fluorescence intensity was measured at a wavelength of 485 nm, *p* < 0.01, analyzed by one-way ANOVA, *n* = 5.

ThT is a fluorescent dye of benzothiazole salts that can selectively bind to β-rich structures, so it is commonly used as a method to detect the degree of amyloid fibril formation [22,23,24]. Based on the results of molecular dynamics analysis of the interaction between tripeptides and Aβ42, WRR and ERW that could potentially inhibit the aggregation of Aβ42 were selected, and the inhibitory effects were further confirmed by ThT fluorescence assay. The fluorescence intensity of the Aβ42 control group and the WRR and ERW co-incubation groups were detected to evaluate the inhibitory effects of WRR and ERW on Aβ42 aggregation. As shown in Figure 4, after Aβ42 alone was incubated at 37 °C for 48 h, it accumulated a large quantity of ThT, the fluorescence intensity of this group was the highest. The fluorescence intensity was dramatically reduced in the WRR- and ERW-treated groups, indicating a decrease in the amount of aggregated Aβ42, showing that either WRR or ERW may effectively prevent Aβ42 polymerization.

### 3.5. Transmission Electron Microscopy Observations of the Effect of WRR or EWR on Aβ42 Aggregation

**Figure 5:**
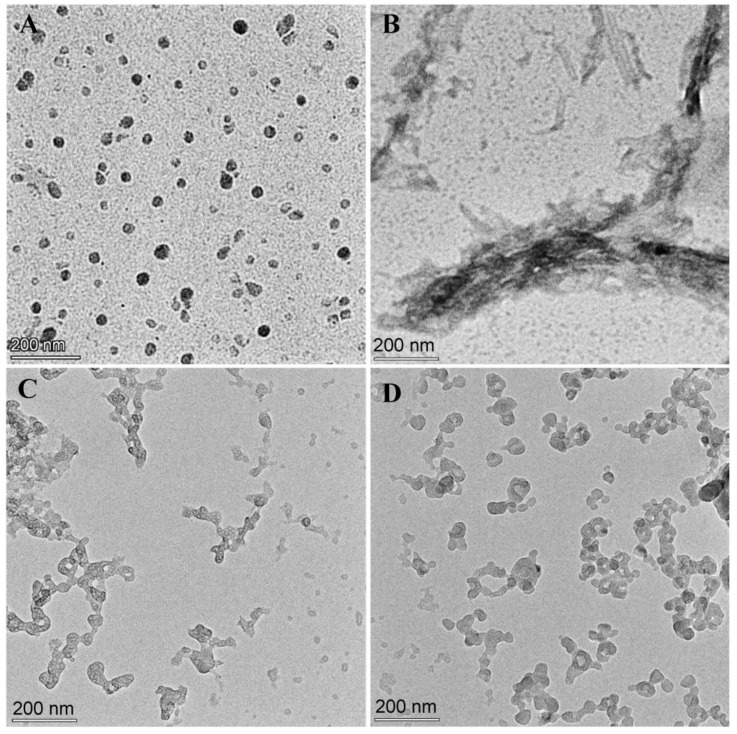
Effect of WRR or ERW on the aggregation morphology of Aβ42 observed using a transmission electron microscope: (**A**) Microscopic image of 10 μM Aβ42 before incubation; (**B**) microscopic image of 10 μM Aβ42 incubated alone for 48 h; (**C**) microscopic image of 10 μM Aβ42 and 40 μM WRR co-incubated for 48 h; (**D**) microscopic image of 10 μM Aβ42 and 40 μM ERW co-incubated for 48 h.

The effect of WRR or ER on the aggregation of Aβ42 was observed with a transmission electron microscope. After incubating Aβ42 for 48 h, plenty of fibrils were plainly visible (Figure 5B). Aβ42 incubated for the same amount of time with WRR or ERW exhibited tiny amorphous aggregate particles (Figure 5C,D). The transmission electron microscopy investigations revealed that the tripeptides inhibited the production of Aβ42 fibrils to some extent, which did not grow into substantial deposits.

### 3.6. Effect of Either WRR or ERW on Cell Death Induced by Secreted Aβ42

**Figure 6:**
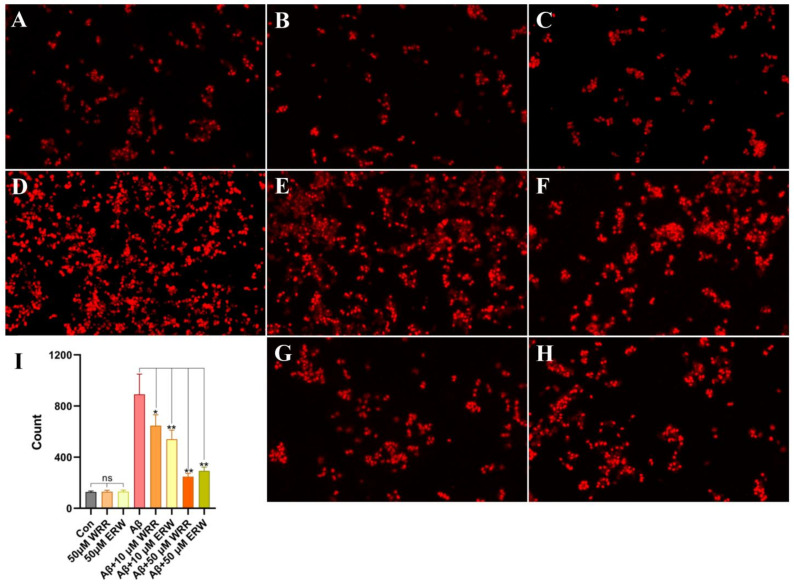
Protective effect of either WRR or ERW on SH-SY5Y cell death induced by secreted Aβ42: (**A**) Status of SH-SY5Y cells not secreting Aβ42; (**B**) status of SH-SY5Y cells secreting Aβ42; (**C**) effects of WRR at 50 μM on the status of SH-SY5Y cells not secreting Aβ42; (**D**) status of SH-SY5Y cells secreting Aβ42; (**E**) effects of WRR at 10 μM on the status of SH-SY5Y cells secreting Aβ42; (**F**) effects of ERW at 10 μM on the status of SH-SY5Y cells secreting Aβ42; (**G**) effects of WRR at 50 μM on the status of SH-SY5Y cells secreting Aβ42; (**H**) effects of ERW at 50 μM on the status of SH-SY5Y cells secreting Aβ422; (**I**) quantification of the effect of either WRR or ERW on the status of SH-SY5Y cells secreting Aβ42. Results are expressed as mean ± SEM. * *p* < 0.05 and ** *p* < 0.01, analyzed by one-way ANOVA, followed by a modified Tukey’s post hoc multiple comparison test, *n* = 3; ns, not significant.

Using a fluorescent microscope, the viability results of Aβ42-secreting SH-SY5Y cells in the control group and the tripeptide treatment groups are presented in Figure 6. Ethidium bromide can incorporate into the DNA of dead cells that have lost their membrane integrity, resulting in the red-stained cells. The morphology of transfected SH-SY5Y cells treated with either WRR or ERW was not different from the negative control, indicating that they had no obvious cytotoxic effects on SH-SY5Y cells. As can be seen in Figure 6D, a large number of SH-SY5Y cells that were transfected and secreted Aβ42 died, which may be due to Aβ42 protein aggregation and neurotoxicity. SH-SY5Y cells treated with 10 μM of the tripeptides showed improved cell survival (Figure 6E,F). With the treatment of 50 μM tripeptides, cell survival was almost unaffected by Aβ42 secretion (Figure 6G,H), indicating that either WRR or ERW could well ameliorate the cytotoxicity of Aβ42 secreted from SH-SY5Y cells.

### 3.7. Effect of Either WRR or ERW on Reactive Oxygen Species Produced by SH-SY5Y Cells Secreting Aβ42

**Figure 7:**
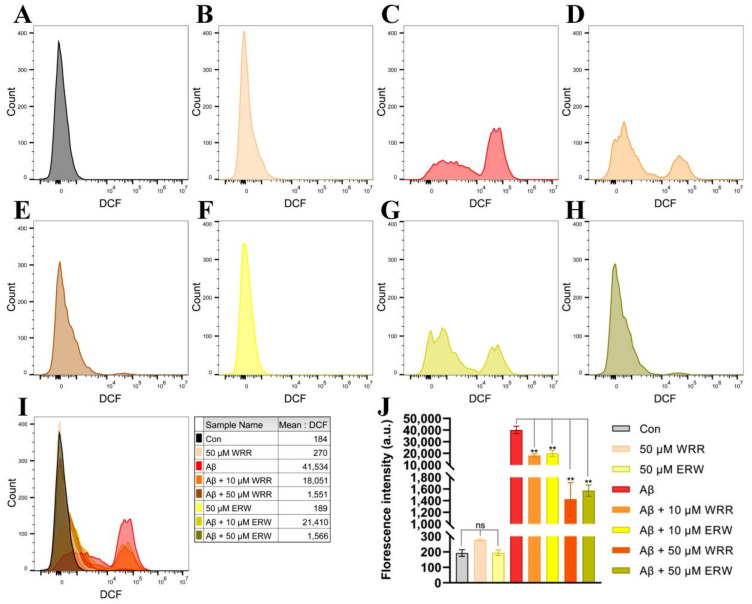
Effect of either WRR or ERW on reactive oxygen species (ROS) levels in SH-SY5Y cells secreting Aβ42: (**A**) ROS levels in SH-SY5Y cells not secreting Aβ42 (Con); (**B**) ROS levels in Aβ42-nonsecreting SH-SY5Y cells treated with WRR at a final concentration of 50 µM; (**C**) ROS levels in SH-SY5Y cells secreting Aβ42; (**D**) ROS levels in Aβ42-secreting SH-SY5Y cells treated with WRR at a final concentration of 10 µM; (**E**) ROS levels in Aβ42-secreting SH-SY5Y cells treated with WRR at a final concentration of 50 µM; (**F**) ROS levels in Aβ42-nonsecreting SH-SY5Y cells treated with ERW at a final concentration of 50 µM; (**G**) ROS levels in Aβ42-secreting SH-SY5Y cells treated with ERW at a final concentration of 10 µM; (**H**) ROS levels in Aβ42-secreting SH-SY5Y cells treated with ERW at a final concentration of 50 µM; (**I**) overlay of the above figures; (**J**) quantitation of the above results. The results were expressed as mean ± SD. ns, not significant; ** *p* < 0.01, by one-way ANOVA, followed by a modified Tukey’s multiple comparison test, *n* = 3.

After SH-SY5Y cells secreting or non-secreting Aβ42 were incubated with WRR or ERW for 24 h, the intracellular ROS levels were measured using fluorescent probe DCFH-DA on a flow cytometer. The results showed that the levels of ROS in SH-SY5Y cells expressing secreted Aβ42 were significantly reduced by the treatments with either WRR or ERW compared with the non-treated SH-SY5Y cells secreting Aβ42, and their effects on ROS levels in the SH-SY5Y cells were dose dependent (Figure 7).

### 3.8. Protective Effect of Either WRR or ERW on Cell Apoptosis of SH-SY5Y Cells Secreting Aβ42

**Figure 8:**
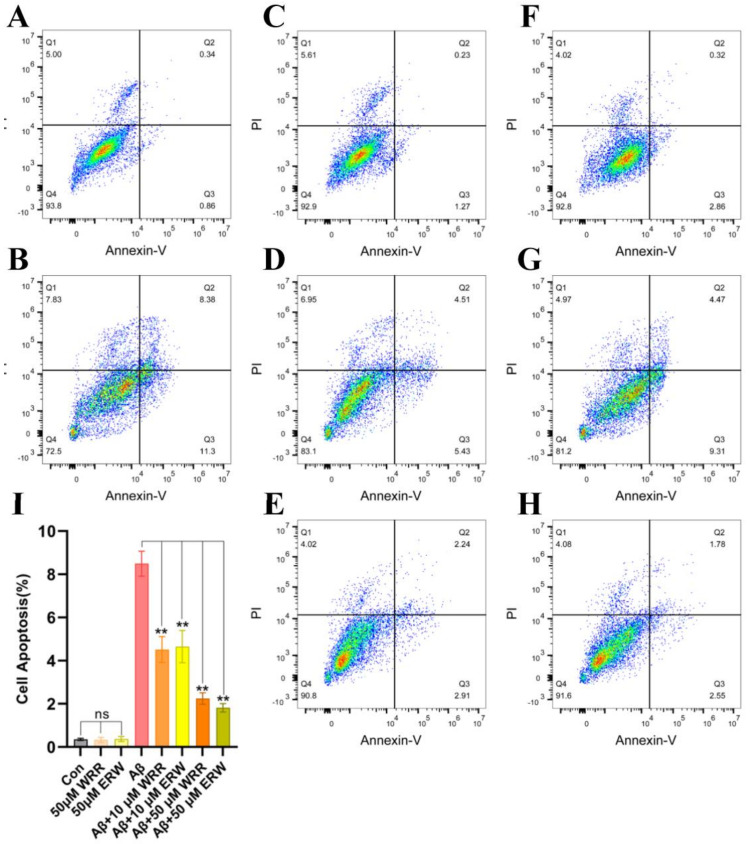
Effect of either WRR or ERW on the apoptosis in SH−SY5Y cells secreting Aβ42: (**A**) apoptosis in Aβ42−nonsecreting SH−SY5Y cells treated with control medium (Con); (**B**) apoptosis in Aβ42−secreting SH−SY5Y cells; (**C**) and (**F**) apoptosis in Aβ42−nonsecreting SH−SY5Y cells treated with WRR at 50 µM; (**D**) and (**G**) apoptosis in Aβ42−secreting SH−SY5Y cells treated with WRR and ERW at 10 µM; (**E**) and (**H**) apoptosis in Aβ42−secreting SH−SY5Y cells treated with WRR and ERW at 50 µM; (**I**) Quantification of cell apoptosis in the above cells. Results are expressed as mean ± SD. ** *p* < 0.01, analyzed by one−way ANOVA, followed by a modified Tukey’s post hoc multiple comparison test, *n* = 3; ns, not significant.

SH-SY5Y cells labeled using apoptosis kit under different treatments were detected by flow cytometry, and the results are shown in Figure 8. Figure 8I shows the quantification of cell apoptosis. In Figure 8, Q1 denotes nude nucleus necrotic cells, Q2 represents necrotic and late apoptotic cells, Q3 represents early apoptotic cells, and Q4 is living cells. As can be seen from the quantitative results of apoptosis in Figure 8I, the additions of either WRR or ERW in the drug administration group can significantly reduce cell apoptosis, especially when the drug administration concentration reaches 50 μM, and the number of apoptosis in the drug treatment group is statistically significant compared with that in the control group without tripeptides. Both WRR and ERW can effectively inhibit the protection of cells from injury, that is, both WRR and ERW can inhibit the aggregation of Aβ42 produced by exocrine expression, and can reduce the damage of SH-SY5Y cells caused by the neurotoxicity of Aβ42.

## 4. Discussion

With the in-depth study of the pathogenesis of Alzheimer’s disease, the development of novel probes and selective inhibitors, including antibodies and small molecule inhibitors, has been promoted, some of which have been successfully used in quantitative, detection or clinical trials [25]. Amyloid accumulation is a key factor in the cause of Alzheimer’s disease. In recent years, great progress has been made in studies targeting Tau protein phosphorylation and metal chelation; abnormal acetylcholinergic energic functions have been proposed, such as a naturally occurring metal sequestrated tripeptide (GHK) and a multifunctional peptide-like inhibitor (P6) of Aβ aggregation inhibitors, which interact with Aβ and prevent the formation of its toxic forms [26]. The metal-chelating agent, GGH, has been shown to selectively chelate Cu^2+^ from the Aβ–Cu complex and improves the survival rate of PC-12 cells [27], but there has been no breakthrough in the research and development of new medicines for Alzheimer’s disease. The amyloid precursor protein has been hydrolyzed and cleaved by β-secretase and γ-secretase successively to produce Aβ42. There are several types of enzyme inhibitors developed for Aβ42, such as γ-secretase inhibitors, β-secretase inhibitors, and Aβ aggregation inhibitors [28]. Some natural compounds and their derivatives to chelate metal ions and reduce neuroinflammation may have good biocompatibility and biosafety. In some molecular dynamics analyses and in vivo experiments, β-folding destructors such as LPFFD and KLVFF have been found to play effective roles in inhibition of the fibrillogenesis of Aβ [29,30]. In addition, some studies have shown that arginine-rich tripeptides have relatively significant inhibitory activities on the formation of Aβ42 oligomers and fibril [13].

In recent years, Aβ42 aggregation inhibitors that are peptide based have attracted more attention in treating AD, but systematic research approaches have seldom been used. Therefore, it is necessary to explore a rapid screening method for developing aggregation inhibitors for treating AD and other diseases. In this study, a fresh approach to the development of medicine was established as such, combining in silico and in vitro research validation for high-throughput screening of drugs for treating AD. Using a self-constructed oligopeptide library to conduct molecular docking and a molecular dynamics analysis, two tripeptides, i.e., WRR and ERW, were screened to have the best potential inhibitory effects.

Oligopeptides can form multiple hydrogen bonds with amino acid residues of Aβ42 at different sites. Chemical groups are constantly moving and interacting with one another due to thermal movement, which makes molecular docking vulnerable because it only identifies certain binding conformations without taking into consideration time. The balance between three interactions, i.e., solute–water, water–water, and solute–solute interactions, determines the final result, which corresponds to the lowest Gibbs free energy in an isobaric system. In this study, a molecular dynamics analysis was used to examine both the binding free energy in protein complexes as well as the time-dependent changes in the locations of the heavy atoms of the ligands attached to Aβ42. We recently published an article [15] in which we proposed thermodynamic mechanisms and a possible pathway for Aβ42 aggregation. Folding and aggregation were driven by a system’s free energy. A per-residue binding free energy analysis highlighted the significant contribution of Phe19 and Glu22 of the Aβ42 monomer in binding with D-enantiomeric RTHLVFFARK-NH2 (rk10) [31], and Phe4, Leu17, Phe20, Ala21, Ala30, Ile31, Leu34, and Ile41 residues of Aβ42 participated in binding with BTT [32] and prevented the formation of β-sheet rich structures of Aβ42 monomer. In the presence of zinc ions [33] and 6N (di-triazole based compound) [34], the α-helix structure of Aβ has been enhanced, while the formation of β-sheet has been prevented, and the natural structure of α-helix has been protected, thus the aggregation of the Aβ42 monomer and disaggregates of Aβ42 protofibrils are prevented. While in our experiment, Aβ42 residues involved in the ERW hydrogen bond interaction were Val18, Phe19, IIe32, Leu34, and Met35. The Aβ42 residues involved in the hydrogen bond interaction between WRR and Aβ42 are 4Phe, 30Ala, and 35Met (Figure 1A,E). The monomer of Aβ42 is globular in nature. However, upon polymerization, more than five Aβ42 strands bind together to form a stable S-shaped structure composed of parallel strands. This structure serves as a frame core for additional polymerization, and the binding free energy between parallel Aβ42 strands in the S-shaped polymer is reduced by arginine dipeptide bound to the hydrophobic regions of an Aβ42 strand [15]. The tripeptides bind to the same hydrophobic regions as those for arginine dipeptide, thereby enabling them to block and reduce the binding free energy between Aβ42 strands. In the current paper, three Aβ42 strands (the trimer displayed in the publication) were taken from the pentamer of Aβ42, with the position of the backbone atoms of all Aβ42 β-strands being restrained during the molecular dynamics analysis to ensure that the conformation of the Aβ42 strands was not altered. The minimum variance of the RMSD values implies they both bind strongly to Aβ42 in some specific conformations to create the most stable complex structure. In addition, the IE values of the complexes are the most negative indicating the strongest attraction between Aβ42 and WRR or ERW. Greater binding free energy is reflected in the bigger changes in Gibbs free energy that occur upon separating either WRR or ERW from Aβ42. The actual inhibitory effects of both WRR and ERW were screened in silico on Aβ42 and were verified in the in vitro experiments. The ThT assay and transmission electron microscopy observations showed that both WRR and ERW can effectively inhibit the aggregation of Aβ42. The fluorescent staining, MTT, and flow cytometric experiments further showed the effects of the two oligopeptides on the cytotoxicity of Aβ42.

## 5. Conclusions

Experiments using molecular docking, molecular dynamics, and molecular pharmacology in the present study have shown that both WRR and ERW not only prevent Aβ42 from aggregating but also attenuate its associated neurotoxicity. The two oligopeptides could be used to create novel medications or food additives that would prevent AD.

## 6. Patents

A patent related to the study is pending.

## Data Availability

All data are available upon request.
